# Family orientation group as a strategy for care in chemical codependency

**DOI:** 10.17533/udea.iee.v37n3e08

**Published:** 2019-10-23

**Authors:** Michele Peixoto da Silva, Adriane Maria Netto de Oliveira, Priscila Arruda da Silva, Simone Algeri, Maria Cristina Flores Soares

**Affiliations:** 1 Social Worker, Master. Rio Grande City Hall, RS - Brazil.Email: chele.p@hotmail.com Rio Grande City Hall Brazil chele.p@hotmail.com; 2 PhD in Nursing. Professor of the nursing school of the Federal University of Rio Grande do Sul, Brazil. Email: adrianenet@vetorial.net Universidade Federal do Rio Grande do Sul Federal University of Rio Grande do Sul Brazil adrianenet@vetorial.net; 3 Nurse, PhD. Federal University of Rio Grande, RS - Brazil. Junior postdoctoral fellow/CNPQ. Email: patitaarruda@yahoo.com.br Fundação Universidade Federal do Rio Grande Federal University of Rio Grande Brazil patitaarruda@yahoo.com.br; 4 Nurse, PhD. Professor. Federal University of Rio Grande, Porto Alegre, RS - Brazil. Email: simone.algeri@gmail.com Fundação Universidade Federal do Rio Grande Federal University of Rio Grande Porto Alegre Brazil simone.algeri@gmail.com; 5 Physiotherapist, PhD. Federal University of Rio Grande, Rio Grande, RS - Brazil. Email: mcflores01@gmail.com Fundação Universidade Federal do Rio Grande Federal University of Rio Grande Rio Grande Brazil mcflores01@gmail.com

**Keywords:** family, substance-related disorders, codependency (psychology), therapeutic community, family therapy, communication, qualitative research., familia, trastornos relacionados con sustancias, codependencia (psicología), comunidad terapêutica, terapia familiar, comunicación, investigación cualitativa.

## Abstract

**Objective.:**

To determine the importance of the family support group in the treatment of codependency, based in reports made by relatives of a therapeutic community for drug addicts.

**Methods.:**

Study conducted in a therapeutic community for alcohol and other drug addicts in a city in the southern extreme of Brazil. This is a qualitative, exploratory and descriptive research with eight drug addict relatives. Data collection occurred through semi-structured interviews and the use of a field diary, focusing on the family orientation group as a space for the health promotion of family members of psychoactive substance users.

**Results.:**

Through discursive analysis, it was found that the family orientation group constitutes an important tool of educational character, capable of responding to the family demands of chemical dependence, as well as rethinking and modifying attitudes and characteristic behaviors of co-dependence.

**Conclusion.:**

The support group is fundamental as a care strategy for codependent families, and may act more effectively when professionals are trained to intervene in the phenomenon of codependency.

## Resumen

**Objetivo.:**

Conocer, a partir del relato de familiares de una comunidad terapéutica para dependientes químicos, la importancia del grupo de apoyo familiar en el tratamiento de la codependencia.

**Métodos.:**

Este estudio se realizó en una comunidad terapéutica para consumidores de alcohol y otras sustancias en una ciudad del extremo sur en Brasil. Se trata de una investigación cualitativa, descriptiva, con la participación de ocho familiares de dependientes químicos. La recolección de datos se realizó con entrevista semiestructurada y con el uso del diario de campo, teniendo como foco el grupo de orientación familiar como un espacio para la promoción de la salud de familiares de usuarios de sustancias psicoactivas.

**Resultados.:**

En el análisis discursivo, se constató que el Grupo de Orientación Familiar es una importante herramienta de carácter educativo, capaz de responder a las demandas familiares sobre la dependencia química, así como repensar y modificar actitudes y comportamientos característicos de la codependencia.

**Conclusión.:**

El apoyo grupal es fundamental como estrategia de cuidado a las familias codependientes y puede ser aún más eficaz cuando los profesionales son capacitados para intervenir el fenómeno de la codependencia.

## Introduction

Dependence on psychoactive substances is a phenomenon that affects not only the user, but also the family environment, causing changes in the routine and behavior of family members. Given the dysfunctional relationship that is established, many families are unable to disentangle themselves from the suffering caused by chemical dependency, becoming what we call codependents. Codependency is defined as a set of pathological patterns, behaviors and thoughts, characteristic of family members or people living directly with drug addicts, which lead to psychological distress.(1,2 ) Although there is no definition of codependency in the DSM-5 and ICD-10, the literature has shown us that chemical dependence may lead family members to illness.(3,4) Therefore, because it is a problem that translates into suffering for the codependent's life, it is important to consider that he / she also needs professional care, since it significantly changes his or her lifestyle, not only with regard to the interaction with the user, but also in relation to other people.(5)

Focusing on professional care on drug users only, fragments and hinders the effective treatment of chemical dependence. Codependents condition their lives around the drug user and, as a result, they no longer have a life of their own. In this context, the family that should be an important ally in the treatment of drug addicts, can no longer support and care them, since it is also suffering a pathological process, having unconsciously allowed the deepening of pathological and dysfunctional relationships. Therefore, by recognizing the family, as a social group that may be sick, highlights the need for social programs. Drug use by one of the family members invariably reveals the fragility of family dynamics. Such vulnerability shows the need to include this social group into treatment, not only as those who have socially defined roles and functions and who may not be able to perform them. The drug addict family must be inserted in the practice of care related to chemical dependence.(6) Despite the increase in the number of studies on this subject, initiatives that give recognition to this family member as an individual who also requires help are incipient, a fact that justifies an important knowledge gap, highlighting the need for greater scientific production on this subject.

The family has been using the health care network to counter the effect (physical and mental symptoms) of the dependent and not the cause (behavior towards the dependent). This behavior may reveal a disease and consequently compromise the quality of life of family members, a fact that justifies the relevance of the study. Health services, in turn, provide care to family members, focusing their approach on drug addicts; there is no actions directed to family members who are codependents. Thus, the family guidance group comes to promote health to those family members who are sick due to chemical dependency. 

This study aims to know, from reports made by family members who are part of a therapeutic community for drug addicts, the importance of the family orientation group (FOG) in the treatment of codependency. This is a study whose approach seeks to characterize the FOG as an educational and participatory space, standing out as a key element for health promotion.

## Methods

A qualitative, exploratory and descriptive study developed with relatives of drug addicts under treatment, in a therapeutic community of southern Brazil. The studied case describes the history lived by families, based on phenomena such as chemical dependence, composing facts, data and information, focusing on the author's final objective, which is to show these data and to evidence what they caused in family functioning over the course of time. 

From the point of view of its purpose, the present study can be classified as exploratory, since studies that contemplate drug addicts do not consider the possibility of family illness. This gap justified the need to know the manifestations of codependency in family members of drug addicts. The research was developed in the FOG of a therapeutic community, which is responsible for the treatment of male drug addicts, over 18 years old. This community is located in the southern extreme of Brazil, in the municipality of Rio Grande / RS, which, according to data from the Brazilian Institute of Geography and Statistics, has a population of approximately 200 000.(7) It is a city port located between Lagoa Mirim, Lagoa dos Patos (the largest lagoon in Brazil) and the Atlantic Ocean. Its channel is the only link between the maritime and inland navigation routes of the state.

Following the authorization of the therapeutic community coordinator and the favorable opinion of the Rio Grande Federal University Research Ethics Committee, under the CAEE 52761515.0.0000.5324, a first approach was made in order to know the functioning of the FOG and to present the survey to family members.

In the study participated eight relatives of drug addicts, users of multiple drugs, who were previously identified as codependents, from the literature, the knowledge arising from the researcher's professional practice and the speeches identified during the group. The following inclusion criteria were used: family members of alcohol and other drug addicts, over 18 years old who were attending the therapeutic community family guidance group, who agreed to participate in the study and had characteristics of codependents, such as: denial, shame, guilt, fear, anger and low self-esteem. 

Family members who did not maintain the minimum attendance frequency in 3 monthly meetings were excluded from the study due to the FOG being held once a week. Also were excluded participating family members under 18 years old, as well as those who did not show co-dependency characteristics. Interviews were started with those family members who manifested characteristics of codependency. The FOG during the observation period of the codependent participants had an average number of 31 relatives. During this period, the group addressed situations experienced with the chemical dependent family member, routine, emotional status, family perspectives and past life experiences.

From the reports involving these themes and the observations that the interviewer evaluated as pertinent, it occurred the identification / selection of the family member with codependency characteristics. Participation in the groups occurred during the months of May and June 2017, weekly from 19h to 21h. The type of bond between participants and drug addicts were: father, mother, daughter, sister, wife and paternal aunt. To preserve their identities, study participants were identified by the letter “F” (family member) followed by the interview sequential number. The study participants were asked to sign the informed consent form, after the researcher informed them about the objectives and methodology of the study, clarifying about their freedom to give up at any time, without personal consequences. 

The techniques used were the semi-structured interview and field diaries records, which served to facilitate the identification of codependent families. For the semi-structured interview, a script consisting of twelve guiding questions was used. The script was related to the interviewee's perception of the situation experienced, feelings about the situation of chemical dependence of the family member and how family life has been affected since the problem arose. The interviews lasted an average of one hour and were recorded with the consent of the participant and took place in a private environment in order to allow an approach without external interventions. After transcribing the interviews, the data were subjected to discursive textual analysis, through rigorous reading, in-depth analysis and its deconstruction, highlighting the units of analysis. 

## Results

Regarding the characteristics of family members participating in this study, six were female and two male, aged between twenty-four and fifty-six years old. Family income ranged from one to five minimum wages. Most have incomplete elementary education and 50% of family members live with the drug addict.

**Table 1:** Sample of family members interviewed

Informant relative	Family bond with the dependent	Age	Family income (mínimum wage = R$ 937)	Educational level	Resides with the dependent
F1	Wife	24	2 minimum wages	Complete high school	Yes
F2	Mother	59	1 minimum wage	Incomplete elementary school	Yes
F3	Paternal aunt	67	3 minimum wages	Complete high school	Yes
F4	Mother	48	2 minimum wages	Incomplete elementary school	Yes
F5	Father	56	5 minimum wages	Incomplete elementary school	No
F6	Father	54	1.5 minimum wages	Incomplete elementary school	No
F7	Daughter	34	2 minimum wages	Complete high school	No
F8	Sister	33	2 minimum wages	Incomplete elementary school	No

In the data analysis process, the following category emerged: Importance of the family group as a care strategy for the codependent family member.

The results show that, from the moment the family members participate in the FOG, there is a change in their conduct. All family members who composed this research were emotionally dependent on their sons, daughters, wives, siblings. For the most part, they could not maintain their identity and autonomy and began to live the life of the drug addict. According to family members: "*the support group has helped a lot, we see a change. After I started attending the group I feel good. There is not much discussion at home today. Before that, it was from morning to night*” (F2).

Considered as a health-promoting and beneficial space for support seekers, F2 seems to have changed her behavior from the moment he discovered that her attention was focused solely on his son, who had been causing constant family quarrels with the support from FOG, F2 can discover new ways for building healthy relationships with the dependent family member. The conversation shows that over time, she noticed improvements in her mental health and some personal growth, which probably reflected in the quality of self-care, revealing the reorganization of family dynamics in order to break with the vicious cycle of a dysfunctional system. 

To F3, the problem experienced by the family member, affects the other relatives and, therefore, need follow-up: “*I think that if everyone who lives at home attended the meetings, it would be better*” (F3 - paternal aunt). The statement presented, reinforces the idea that the behavior manifested towards the dependent may represent a disease and, consequently, compromise the quality of life of all family members.

When the family member seeks help to break the dysfunctional cycle, it is usually in the support services that they find it. However, they do not always seek to modify their behavior towards the dependent. It is observed in the statements below that family members seek the FOG as a “distress call”, when they realize that chemical dependence has caused them suffering. F6 can reevaluate feelings, needs, reestablish limits that until then, he denied due to the dependence of the child. F7, rescued values that until then, he had lost in his role as a father. F8, by recognizing herself as a codependent, was able to better address his brother's drug addiction problem in a healthy and proactive manner: "*The group has helped us in what we need, for example, to get closer, talk more, learn more about our own family…*” (F6 - Father); “My life before I became codependent on my mother was quiet, I was a happy person, I was not ashamed*. Then I started to be ashamed, I didn't finish what I had to*... If it wasn't for this group help, I wouldn´t feel as good and calm as I am now in this interview” (F7 - Daughter); “*I consider myself a codependent because I am living his life, leading my brother's life. Certainly, the group helps us by learning how to help and to support my brother without harming me*” (F8 - Sister).

The codependent needs to realize and acknowledge that, its relationship with the drug addict is pathological, and that its efforts to help and protect him are doomed to failure, due to their inability to care because they are also ill. Therefore, it is clear that with the help of the group, the family member can identify the difficulties arising from living with the drug addict, admitting that the disease affected him, making him a codependent. Reports show the relevance of family participation in treatment, especially when someone becomes ill due to their family member.

## Discussion

The results allow to infer how much families mobilize due to the problem, becoming affected with the behavior of the dependent family member.(8-10) Due to the conflicts experienced by chemical dependence, attempting to overcome these conflicts and seeking for family reorganization, it is that families may find in the family orientation group the care they need.(11,12) It should be noted that the problem experienced by a family member usually affects the other family members, shifting the perception sense of individuality; even if each one tries to maintain it, it is difficult to do so, due to the bond that permeates these relationships,(13) corroborating F3's statement. 

In the process of illness, family members experience situations such as anguish, conflicts, fears, doubts. Therefore, they require a therapeutic space to be heard and helped.(4) That is why the treatment should be systematic, because if the family fails the drug user will fail as well. Because of this lack of support for families, many of them become codependent on drug addicts.(14) Recognition of every individual's limitations is essential for the health restoration and the pursuit of quality of life. However, to obtain an effective result, professionals need to be able to address the phenomenon of codependency, taking into account the family's life history and the aspects involved, in order to better understand family functioning.(15,16) It is important to emphasize the fact that, when the codependent makes himself more available to others than to himself, creates a permissive profile that, in most cases, prevents the drug user from assuming responsibility for its actions. This difficulty recognizing the disease, and therefore the drug user does not seek treatment.(3)

As limitations of the study, it is worth mentioning that the research was conducted just at one location, not being explored in other units, such as the psychosocial care centers and other therapeutic communities provided with health professionals. However, the study drew attention to the rethinking of professional practices and nursing / health research, since family codependency is present in all spaces. Still, the focus is concentrated on the dependent.

The study sought to highlight the importance of the support group as a care strategy for codependent families. This may act more effectively when professionals are trained to intervene in the phenomenon of codependency. As indicative for the practice of nursing/health, the findings provide subsidies that may contribute to the elaboration of intervention strategies, subsidizing the identification of family codependency, the proposition of solutions and decision making, such as the creation of public policies directed to the researched profiles.

The support group is recognized as a protection space. Families achieved, throughout the meetings, to understand that their life was dependent on the drug user and so, they no longer lived their own life. The characteristics presented by the families, further reinforced how exposed and vulnerable they were to the dependent. Thus, when dealing with a disease that translates into suffering for the codependent's life, as for the addict, this study becomes relevant for the scientific community, still little explored in the academic environment and in health services. 

The family uses health services, often to alleviate the physical and mental symptoms arising from the imbalance of the family system. However, health professionals need to be equipped to identify what leads families into this disease. Although health services offer attention directed to family members, their approach is still focused on the dependent, which justifies the importance of the study.
